# Age estimation by pulp/tooth area ratio in anterior teeth using cone-beam computed tomography: comparison of four teeth

**DOI:** 10.1590/1678-7757-2018-0722

**Published:** 2019-07-25

**Authors:** Sina HAGHANIFAR, Fateme GHOBADI, Nazmehr VAHDANI, Ali BIJANI

**Affiliations:** 1 Babol University of Medical Sciences Babol University of Medical Sciences Health Research Institute Oral Health Research Center Babol Iran Babol University of Medical Sciences, Health Research Institute, Oral Health Research Center, Babol. Iran.; 2 Babol University of Medical Sciences Babol University of Medical Sciences Student Research Committee Babol Iran Babol University of Medical Sciences, Student Research Committee, Postgraduate student of oral and maxillofacial radiology, Babol, Iran.; 3 University of Medical Sciences University of Medical Sciences Health Research Institute Social Determinants of Health Research Center Babol Iran University of Medical Sciences, Health Research Institute, Social Determinants of Health Research Center, Babol, Iran.

**Keywords:** Cone-beam computed tomography, Dental pulp cavity, Forensic dentistry, Incisor, Tooth

## Abstract

**Objectives:**

Age estimation is one of the most important factors in forensic medicine. Measuring secondary dentin deposition using cone-beam computed tomography images is an easy and noninvasive method. The aim of this study was to evaluate cone-beam computed tomography images as a reliable method to estimate chronological age by pulp/teeth ratio in anterior teeth in Iranian population.

**Methodology:**

A total of 649 CBCT images from 377 Iranian patients aged between 20 and 69 years were evaluated. Pulp/teeth ratio (PTR) in maxillary and mandibular canine and central incisor teeth was measured in the axial and sagittal sections using OnDemand 3D Dental software. The Pearson correlation coefficient was determined to evaluate the correlation between the variables. Linear regression analysis, as well as age estimation formula, was used for each tooth separately.

**Results:**

The regression analyses indicated that maxillary central incisors were more reliable for age estimation (R^2^=0.586 and standard error of estimate=7.045) compared with the other anterior teeth studied. Maxillary canine teeth had the lowest predictive power (R^2^=0.392 and standard error of estimate=8.387). Also, comparison of the axial and sagittal sections showed that the axial sections had a higher predictive power. (R^2^=0.48 for axial plans and R^2^=0.328 for sagittal plans)

**Conclusion:**

The use of cone-beam computed tomography in age estimation by pulp/teeth ratio of anterior teeth is useful and a reliable method for age estimation in Iranian population.

## Introduction

Age estimation is one of the most important issues in forensic medicine and archeology.^1^ Age is one of the three indices of living creatures used to identify unidentifiable bodies such as those affected by accidents and natural disasters.^2 , 3^

Age estimation in adults is more difficult and challenging compared with that of children, since dental and skeletal growth are completed in adults. The methods used for adults based on the skeletal degenerative changes^4 - 8^ are not as accurate as those used for children, with focus on dental development such as dental hard tissue mineralization and dental eruption.^9^

The dental tissue is the hardest tissue in the human body, is highly resistant to physicochemical damages and can preserve its structure for a long time after death.^10 , 11^ There are different dental-based age estimation methods.^12 - 16^ Most of these methods are time-consuming, demand expensive laboratory equipment and require dental extraction. Hence, these methods are not applicable to people who are alive nor to bodies without legal authorization for tissue harvesting.

After tooth eruption in the oral cavity, secondary dentin deposition starts in the teeth and continues to the end of life, gradually reducing the size of the pulp chamber.^17 , 18^ Gustafson^19^ (1950) introduced the secondary dentin deposition as an index for age estimation. There is a direct relationship between the secondary dentin deposition and age; hence, it can be used as a factor to predict and estimate the age.^20 , 21^

Application of radiographic images for age estimation by measuring the pulp chamber size and the amount of secondary dentin deposition is an easy, noninvasive method, applicable to both living and dead people with no need for tooth extraction. The study by Kvaal, et al.^21^ (1995) was one of the first investigations that developed an indirect age estimation method based on the amount of secondary dentin deposition using radiographic images and suggested different methods for longitudinal and transverse measurement of the teeth. Cameriere, et al.^22 - 24^ (2004) also introduced a similar age estimation method based on pulp/tooth ratio (PTR) using 2-dimensional (2D) radiographic images such as periapical and panoramic ones. Data analysis is not possible in 2D images of teeth with crowding, rotation or superimposition. Also, using these images, the measurement is only possible in the mesiodistal but not in the buccolingual dimension.^25^ In recent years, new advancements in 3-dimensional (3D) imaging technologies, such as cone-beam computed tomography (CBCT), enabled dentists to use the images in the evaluation of the correlation between age and size of the pulp chamber. Several researchers estimated age by using three-dimensional cone-beam CT to overcome the limitation of 2D images and concluded that measuring the pulp is a useful indicator for age.^1 , 3 , 26 - 28^

The purpose of this study was to use images of the maxillary and mandibular canine and central incisor for chronological age estimation and to establish age estimation models from CBCT sagittal and axial sectional images using a numerical analysis software in the Iranian population and also, to compare the predictive power of the four anterior teeth.

## Methodology

### Subject selection

This study was approved by ethics committee of Babol University of Medical Sciences (Grant No. 9644720 and ethics code: mubabol.rec.1396.66)

With a confidence interval of 95% and power of 90% to obtain a correlation confidence of 0.3 between PTR and chronological age, 100 samples were estimated for each type of tooth. However, this study considered more than 100 samples for each type of tooth for better accuracy.

The sample of the study consisted of 649 CBCT images of anterior teeth, including 153 maxillary central incisors and 150 maxillary canine teeth, 150 mandibular central incisors and 196 mandibular canine teeth of 377 Iranian patients (220 females and 157 males) aged between 20 and 69 years, with mean age of 38.9± years. The samples were selected from the population of patients who visited a private clinic of oral and maxillofacial radiology between December 2015 and December 2017. The CBCT images used in this study were taken for routine therapeutic treatment and clinical evaluation. Therefore, informed consent was not required.

The inclusion criteria were the availability of maxillary or mandibular central incisor or canine tooth images and the exclusion criteria were any sign of dental caries, dental restorations, root canal treatment, excessive tooth wear, internal or external teeth resorption and any other conditions that aﬀected the pulp/tooth area or dental formation, such as systemic conditions and syndromes.

### CBCT image acquisition

All CBCT images used in this study were obtained using Cranex 3D machine (Soredex, Helsinki, Finland). The exposure parameters for the CBCT images were 89 kVp, 6 mA and 6×8 cm field of view (FOV).

The images were stored in DICOM format and to reconstruct them, OnDemand 3D Dental^TM^ software (Cybermed Co., Tustin, USA) was employed. This software was used to determine the tooth and pulp areas in the axial and sagittal sections.

## Methods

All evaluations were conducted by an observer (oral and maxillofacial radiologist) in a semi-dark room by a 23.8-inch, Color Depth bit 24, LCD monitor (Dell, China).

After opening the appropriate image using OnDemand 3D Dental^TM^ software; the brightness, contrast and sharpness of the images were modified, if needed, to get better results.

**Figure 1:**
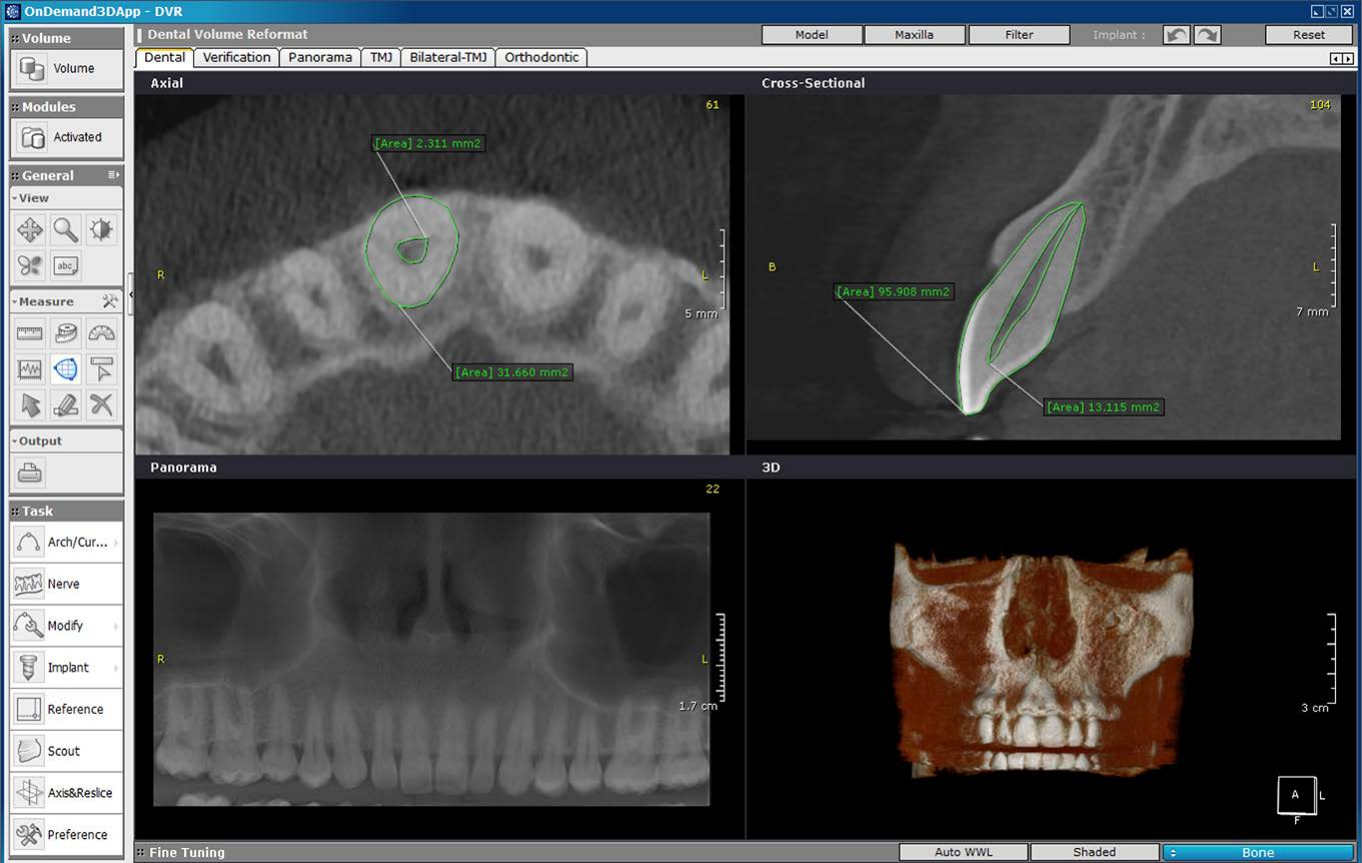
Axial and sagittal views of the maxillary central incisor from CBCT seen in OnDemand 3D Dental program. The outer line delineate the area of the tooth. The inner line delineate the area of the pulp

To determine the pulp/teeth ratio (PTR) in the sagittal plan, first, the longitudinal axis of the tooth from the crown tip to root apex was determined and then the best midsagittal section with the largest tooth and pulp areas was selected by scrolling the image on the cross sectional plan of the software. The tooth area was measured using the area tool in the tool bar of OnDemand 3D Dental^TM^ software. A set of points was used to determine the entire tooth outlines and after completion of the tracing, the area was shown in the frame. Next, the outline of the pulp from pulp chamber in the crown to root apex was traced and the area of the pulp was measured similarly ( Figure 1 ). Finally, PTR_sagittal_ was measured by dividing the area of the pulp into the area of the tooth.

To determine PTR in the axial plan, first, the longitudinal axis of the tooth from the crown tip to root apex was determined and then, the axial section of the tooth was set at the cementoenamel junction (CEJ) region by scrolling the image at the axial plan of the software. Similar to the sagittal plan, a set of points was used to measure the tooth and pulp areas, and then the PTR_axial_ was calculated.

The tooth and pulp areas were measured in triplicate by the observer and the mean values were considered as the final radiographic measures.

### Statistical analysis

Pearson correlation coefficient was used to evaluate the relationship between the variables, using SPSS, version 17 (SPSS Inc., Chicago, IL, USA). The linear regression analysis was performed for each tooth using PTR based on the sections and sex, and then, the age estimation was separately performed for each tooth. The standard error of estimate (SEE) and the values of coefficient of determination (R^2^) were measured, respectively, to predict the standard deviation of the estimated age compared with the actual age, and to evaluate the predictive power of the variables such as type of tooth and section.

## Results

This study found no statistical significant differences for intra-observer variances (r=0.97 and p=0.485).

The minimum, maximum and mean values of the PTR in different types of teeth in the axial and sagittal sections are shown in Table 1 .

**Table 1:** Descriptive statistics for the pulp to tooth area ratio in anterior teeth

Plan	Tooth	No	Mean	SD	Min	Max
Sagittal	Upper central	153	0.105	0.026	0.05	0.18
	Upper canine	150	0.150	0.030	0.07	0.28
	Lower central	150	0.127	0.034	0.05	0.22
	Lower canine	196	0.162	0.032	0.07	0.26
Axial	Upper central	153	0.046	0.014	0.01	0.08
	Upper canine	150	0.047	0.012	0.02	0.09
	Lower central	150	0.033	0.010	0.01	0.06
	Lower canine	196	0.043	0.012	0.01	0.08

No: Number ,SD: Standard deviation ,Min: Minimum ,Max: Maximum.

**Figure 2:**
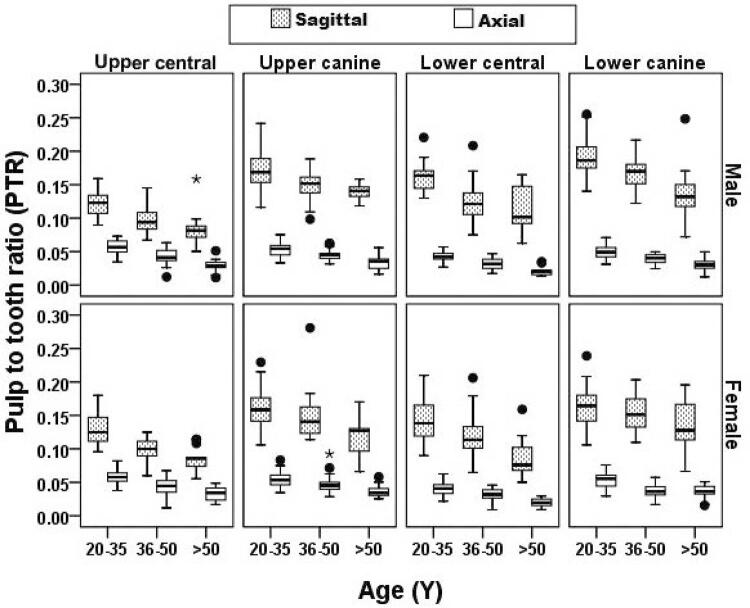
Distribution of pulp to tooth area ratio (PTR) in all teeth by age and sex group

The distribution of the PTR in different ages ranging according to the type of tooth, section and sex is shown in Figure 2 . By increasing the actual age, the PTR in the maxillary and mandibular central incisor and canine teeth in the axial and sagittal sections decreased in both sexes. ( Figure 2 )

The Pearson correlation coefficient between the age and PTR based on type of teeth, type of sections and sex is shown in Table 2 .

**Table 2:** Pearson’s coefficient of correlation between chronological age and physiological ratio

		Male		Female	
		PTR Axial	PTR Sagittal	PTR Axial	PTR Sagittal
Upper Central	r	-0.743	-0.623	-0.710	-0.759
	P	0.000	0.000	0.000	0.000
Upper Canine	r	-0.655	-0.441	-0.553	-0.479
	P	0.000	0.000	0.000	0.000
Lower Central	r	-0.705	-0.533	-0.699	-0.625
	P	0.000	0.000	0.000	0.000
Lower Canine	r	-0.764	-0.624	-0.642	-0.330
	P	0.000	0.000	0.000	0.000

PTR: Pulp to tooth area ratio, r: Correlation coefficient, P: P value.

According to Table 2 , there was a significant correlation between the chronological age and physiological ratio (PTR). The correlation coefficient lower than -0.7 has very strong correlation, between -0.7 and -0.5 has strong correlation, between -0.5 and -0.3 has moderate correlation and higher than -0.3 has weak correlation ( Table 2 ). In this context, the strongest correlation in male subjects was observed in the mandibular canine teeth in axial sections and the strongest correlation in female subjects was observed in the maxillary central teeth in sagittal sections.

The regression equation for age estimation, SEE and the values of coefficient of determination (R square) are shown in Table 3 .

**Table 3:** Regression models using physiological ratios for predicting chronological ages

Plan	Tooth	Gender	Linear Regression Equation	SEE	R^2^
Axial	Upper Central	M	Y=66-543X	7.21	0.551
		F	Y=64.7-524X	7.68	0.504
	Upper Canine	M	Y=67.3-591X	7.98	0.429
		F	Y=61.2-454X	9.05	0.306
	Lower Central	M	Y=65.4-734X	7.74	0.498
		F	Y=61.8-657X	7.40	0.488
	Lower Canine	M	Y=71.5-789X	7.83	0.583
		F	Y=61-535X	8.53	0.412
Sagittal	Upper Central	M	Y=69-264X	8.43	0.388
		F	Y=73.6-309X	7.10	0.576
	Upper Canine	M	Y=66.9-169X	9.47	0.195
		F	Y=63-160X	9.53	0.229
	Lower Central	M	Y=63.7-171X	9.24	0.284
		F	Y=62.5-184X	8.08	0.391
	Lower Canine	M	Y=75.3-216X	9.47	0.389
		F	Y=56.9-127X	10.5	0.109

SEE: Standard error of estimate, R^2^: Coefficient of determination, M: Male, F: Female, Y:predicted age, X: PTR.

**Figure 3:**
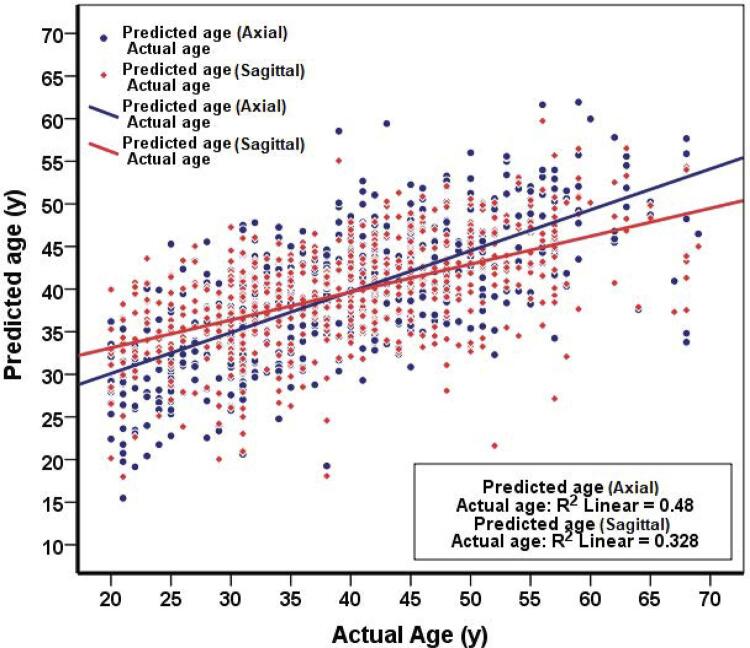
Plots of actual age versus predicted age of all teeth

According to Table 3 , in male subjects, age estimation by lower canine tooth had the highest R^2^ in both sections and in female subjects, age estimation by upper central tooth had the highest R^2^ in both sections. Figure 3 was plotted based on these values: the measured R^2^ for axial and sagittal sections was 0.48 and 0.328, respectively, indicating the highest power of axial sections over the sagittal ones to predict age.

Thereafter, by considering all the involved variables (sex and type of sections) the R^2^ and SEE of each tooth were measured using linear correlation analysis and the results were as follows: R^2^=0.586 and SEE=7.045 for maxillary central incisor; R^2^=0.525 and SEE=7.286 for mandibular central incisor; R^2^=0.509 and SEE=8.107 for mandibular canine teeth and R^2^=0.392 and SEE=8.387 for maxillary canine teeth.

Therefore the highest R^2^ and lowest SEE were observed in the maxillary central incisor, indicating the superiority of this tooth compared with other anterior teeth to estimate the chronological age.

**Figure 4:**
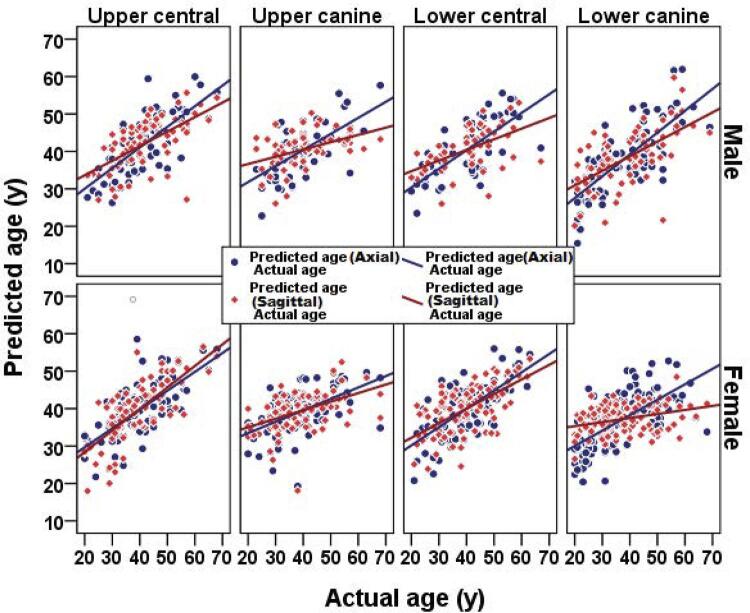
Plots of actual age versus predicted age of upper, lower central and canine teeth

Figure 4 shows the predicted age versus actual age based on the different variables of this study.

## Discussion

CBCT images were used in this study for pulp measurements and age estimation. Such images benefited from controlled magnification compared with the 2D radiography images, such as panoramic and periapical ones. In addition, other advantages of CBCT images compared those those of micro-CT were the lack of superimposition of dental structures, the possibility for 3D analyses, the lack of geometric distortion, the lower radiation dose compared to that of CT, and the greater FOV.

Due to ethical considerations, CBCT images could not be taken from patients. Therefore, the samples were selected by simple sampling from the archive of the private oral and maxillofacial radiology center.

The sagittal and axial sections were choson because the sagittal sections show wide pulp and tooth areas and the highest amount of secondary dentine deposition is observed in the cervical area.^18^

There was a more significant relationship between the age and PTR in the axial section compared to the sagittal section in both sexes; in addition, the predictive power of axial section in age estimation was higher than that of the sagittal section (R^2^=0.48 for axial plans, R^2^=0.328 for sagittal plans). Similar results were also reported by Rai, et al.^3^ (2016) for maxillary canine teeth (r=-0.32 for axial plans, r=-0.11 for sagittal plans). The findings of this study can be explained by the fact that the axial sections (at cervical area) are far from the occlusal stresses and are not affected by secondary dentine deposition due to non-aging causes.^9 , 29^ However, findings of the present study were inconsistent with those of Lee, et al.^25^ (2017) for maxillary canine teeth. In their study, the sagittal sections presented a stronger correlation with age compared with the axial sections (r=-0.65 for axial plans, r=-0.72 for sagittal plans). The reason for this difference can be probably attributed to the studied population, since genetics and other environmental factors can also affect the secondary dentine deposition. In this sense, age estimation equations have higher accuracy if they are used for each population separately.^30^

In this study, the correlation coefficient between PTR_sagittal_ and age in lower canine in both sexes differed (r=-0.624 in males and r=-0.330 in females). The t-test showed that the mean values of PTR_sagittal_ in both sexes was significantly different (p=0.042). Therefore, these differences can be due to the different buccolingual diameter in lower canines between both sexes.

The maxillary and mandibular central incisors and canine teeth were selected in this study because maxillary central incisors have simple anatomical features and are convenient for root canal measurement, and the maxillary and mandibular canine teeth have the longest and most durable roots of the teeth. The mandibular central incisors are among the teeth with low index of caries and remain intact for a long time.

These results showed the most predictive power and lowest standard error of age estimation in the maxillary central incisors (R^2^=0.586), indicating the superiority of these teeth over the others in age estimation. The predictive power of maxillary central incisors measured in this study was similar to that of the study by Wu, et al.^31^ (2016) (R^2^=0.576). In the study of Biuki, et al.^32^ (2017) and Gulsahi, et al.^33^ (2017), as in the present study, maxillary central incisor has the highest predictive power of age estimation. However, the method for their study differs from the present one. In their study, the pulp volume to tooth volume ratio was used for age estimation but in this study, the pulp area to tooth area ratio in axial and sagittal sections was used.

The predictive power of this study was lower than those of Someda, et al.^34^ (2009), (R^2^=0.65–0.77 for mandibular central incisors) and Aboshi, et al.^35^ (2010), (R^2^=0.635 for mandibular first premolar and R^2^=0.703 for second premolar) since they used micro-CT images. This technique has higher spatial resolution and measurement accuracy^36^ , although micro-CT can only be applied to small sizes and not to craniofacial structures.^37^

Based on the data obtained, age estimation in subjects older than 60 was not very reliable. The reason can be attributed to the unjustifiable reduction of pulp size within the age range of 60 to 69 years.^36^ This finding was similar to those of previous studies, indicating that the highest rate of secondary dentin deposition occurs in the adolescence, which is reduced in old age.^35 , 38^

The SEE was less than 10 years old in the current study, which was acceptable in forensic medicine.^39^

Further studies with more than one examiner, larger sample size selection, different types of teeth and multiple regression analysis must be used to improve the accuracy of the study.
